# Investigating the Relative Significance of Drug-Related Problem Categories

**DOI:** 10.3390/pharmacy5020031

**Published:** 2017-06-09

**Authors:** Lene Juel Kjeldsen, Trine Rune Høgh Nielsen, Charlotte Olesen

**Affiliations:** 1 The Danish Research Unit for Hospital Pharmacy, Amgros I/S, 2100 Copenhagen, Denmark; 2 The Hospital Pharmacy, Region Zealand, 4700 Næstved, Denmark; trn@regionsjaelland.dk; 3 The Hospital Pharmacy, Central Denmark Region, 8000 Aarhus, Denmark; CHAOLN@auh.rm.dk

**Keywords:** drug-related problems, clinical significance, review, categorisation systems, hospital

## Abstract

The aim of the review was to investigate whether an assessment of clinical significance can be related to specific drug-related problems (DRPs) and hence may assist in prioritizing individual categories of DRP categorization systems. The literature search using Google Scholar was performed for the period 1990 to 2013 and comprised primary research studies of clinical pharmacy interventions including DRP and clinical significance assessments. Two reviewers assessed the titles, abstracts, and full-text papers individually, and inclusion was determined by consensus. A total of 27 unique publications were included in the review. They had been conducted in 14 different countries and reported a large range of DRPs (71–5948). Five existing DRP categorisation systems were frequently used, and two methods employed to assess clinical significance were frequently reported. The present review could not establish a consistent relation between the DRP categories and the level of clinical significance. However, the categories “ADR” and possibly “Drug interaction” were often associated with an assessed high clinical significance, albeit they were infrequently identified in the studies. Hence, clinical significance assessments do not seem to be useful in prioritizing individual DRPs in the DRP categorization systems. Consequently, it may be necessary to reconsider our current approach for evaluating DRPs.

## 1. Introduction

Drug-related problems (DRPs) are associated with increased morbidity and health care costs, and identifying and addressing DRPs are essential tasks within the health care system [1,2,3]. Various definitions of a DRP exist, and one of them is: “a circumstance related to the patient’s use of a drug that actually or potentially prevents the patient from gaining the intended benefit of the drug” [4].

DRPs may be identified and solved as a part of conducting medication reviews, which in practice, are often performed by clinical pharmacists in collaboration with physicians [5]. When reporting process measures of medication reviews, DRP categorization systems are frequently used. Several DRP categorization systems exist [4,6]. Some have been developed in primary care, where patients are usually an active participant in the medication review, while other DRP categorization systems only target issues that can be identified based on patient charts. The quality of DRP categorization systems has been reviewed by Van Mill et al. [4]. The authors identified 14 DRP categorization systems, but only three had been assessed for their usability in practice and internal validity, and none of the categorization systems met the proposed criteria for an optimal system [4]. Some had integrated the suggested intervention as a part of the DRP categorization system, which has also been reported by other authors [7].

However, none of the DRP categorization systems prioritise the individual categories—or indicate whether some categories are of a higher clinical importance. This information might be of value when implementing interventions addressing DRPs, as well as for evaluation purposes. An example of a DRP, which is frequently associated with a significant clinical importance; hospitalization, is adverse drug reactions (ADRs) [2,3]; however, not all DRP categories may be associated with this clinical outcome. A way of indicating a value to individual DRPs or suggested interventions is by assessing the clinical significance. Various definitions of clinical significance exist, and one of them is “the practical importance of a treatment effect—whether it has a real genuine, palpable, noticeable effect on daily life” [8]. Some clinical significance categorization systems have been developed, mainly as a part of assessing medication errors/patient safety issues [9,10,11]. Such systems have been applied to assess the clinical significance of DRPs, but it is unknown whether the clinical significance categorization systems may be associated with DRP categories.

The aim of this systematic review was to investigate whether an assessment of clinical significance can be related to specific DRPs and hence may assist in prioritising individual categories of DRP categorization systems.

## 2. Materials and Methods

### 2.1. Search Strategy

When conducting our literature search, we sought to identify studies where drug-related problems (DRPs) were identified and categorised, and the clinical significance of the DRPs was assessed. A literature search was performed using the search phrases: A: “drug related problem”, “medicine related problem” and “medication related problem” combined with B: “clinical significance”, “clinical relevance” and “clinical importance” combined with C: “categorisation”, “categorization” and “classification”.

Publications were included if they:

- described primary research
- were published in English
- described interventions delivered by clinical pharmacists

Publications were excluded if they:

- were not published as a research paper (e.g., reviews, books, congress abstracts, posters, reports, protocols)
- did not present data on DRPs
- described selected DRPs only (e.g., drug-drug interactions)
- did not present a DRP-categorization system
- did not present data on an assessment of clinical significance
- presented data for a sub-study, where the original study had been included

The search was performed for the period from 1990 to 2013 using Google Scholar (TRHN). Google Scholar was used to ensure inclusion of the largest possible number of papers, because several studies presenting DRPs were expected to have been published in non-indexed journals. A similar search in PubMed yielded less than five hits.

### 2.2. Assessment

All titles and publication types from the original search were reviewed independently by TRHN and LJK. Subsequently, the abstracts were reviewed by these two authors. Thereafter, full-text articles were reviewed independently by CO and LJK. Finally, CO and LJK extracted data from the studies independently. At every step, disagreements were resolved by consensus. The data extracted were details regarding the study, the intervention, DRP-categorisation, and the clinical significance assessment. Action on DRPs were differentiated between the acceptance rate and implementation rate, because the implementation of a suggested intervention is often dependent on an action by the physician, and even though a physician accepts a suggested intervention, the action of implementing it may be lacking. The clinical significance of DRPs related to medication reconciliation was excluded, since DRPs related to medication reconciliation were not part of the inclusion criteria.

### 2.3. Study Selection

**Figure 1:**
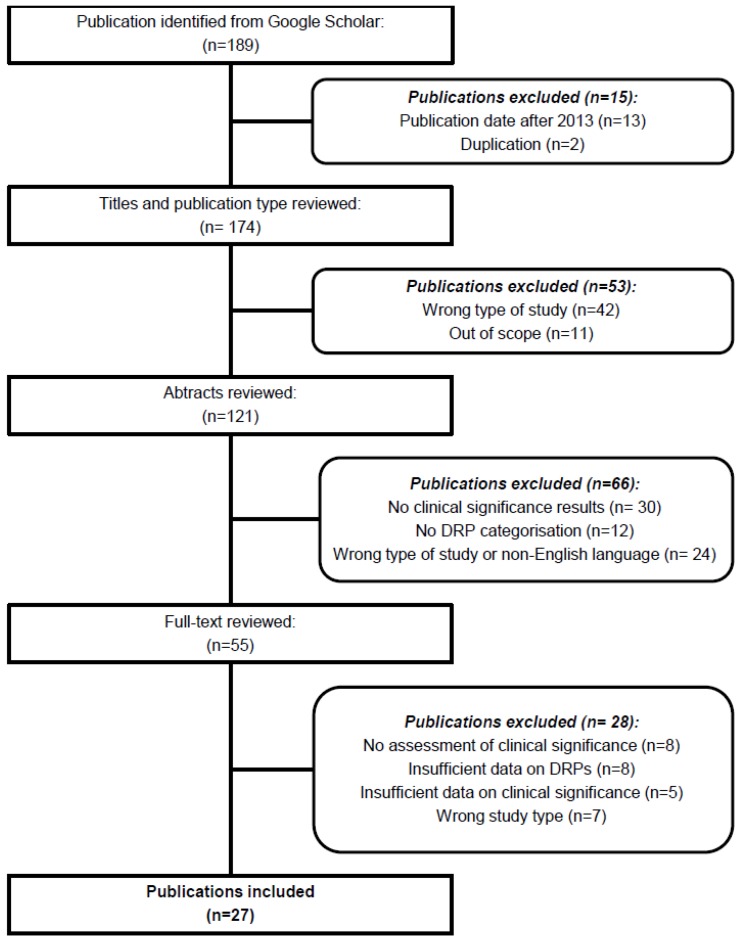
Flow chart of study selection for the review.

A total of 189 studies were identified in the Google Scholar search (Figure 1). After removing 15 papers due to duplicate records and publication dates after 2013, the in- and exclusion criteria were applied to 174 unique publication titles and subsequently to 121 unique abstracts (Figure 1). Of these, 55 full-text publications were reviewed, and 28 were excluded due to: No assessment of clinical significance (*n* = 8) [12,13,14,15,16,17,18,19], Insufficient data on DRPs (*n* = 8) [20,21,22,23,24,25,26,27], Insufficient data on clinical significance (*n* = 5) [28,29,30,31,32], and Wrong study type (*n* = 7) [33,34,35,36,37,38,39]. Finally, 27 unique publications were included in the review [40,41,42,43,44,45,46,47,48,49,50,51,52,53,54,55,56,57,58,59,60,61,62,63,64,65,66].

## 3. Results

### 3.1. Description of Studies

The included studies had been conducted in 14 countries in Europe, Asia, Australasia, Africa, and North America, and most frequently, in India and Australia, with six studies each (Table 1). The majority of the studies were conducted at one hospital (*n* = 17), followed by community pharmacies (*n* = 5) (Table 1). A single study included patients from hospitals in two countries (UK and Saudi Arabia). Patients included in the study ranged from 46 to 737, and only seven studies had included more than 200 patients (Table 1). Most of the studies involved a medication review, either by itself of in combination with other pharmaceutical care activities (Table 1). The description of the interventions varied considerably between the studies and it was not possible to establish the level of similarities among the interventions (i.e., level of patient involvement, nature of collaboration with physicians, data sources used (e.g., laboratory values, chart information), and follow up). The number of DRPs identified varied considerably (71–5948), as well as the acceptance rate (47–97%) and the implementation rate (67–90%); however, most studies did not report a separate implementation rate (*n* = 20) (Table 1).

Several of the studies used DRP categorization systems based on an existing one, but which were adjusted to the conditions of their study (Table 2). Five existing DRP categorization systems were frequently referenced as inspiration: Modified versions of Hepler and Strand [67], Strand [68], Cipolle, Strand and Morley [69], PCNE [70], and DOCUMENT [66], and some studies had applied the DRP categorization systems directly (Table 2). However, some studies did not refer to any published DRP categorization system (*n* = 5). Overall, the number of categories used varied considerably among the studies, ranging from six to 17 categories. No category was used by all DRP categorization systems, but some of the categories were frequently used, e.g., “Untreated indication”, “Improper drug selection”, and “Adverse drug reaction” (Table 2). Several DRP categorization systems used an “Other” category, irrespective of how many other categories the DRP categorization system consisted of. Consequently, a comparison of the frequencies of categorised DRPs in the studies was difficult.

Fewer categories were used to categorise clinical significance (Table 3). In general, three categories were used; “Major, Moderate, and Minor” or similar labels for three categories (Table 3). For the categorization systems where more categories were added (Extremely important, low, insignificant, and adverse significant), the three core categories covered the vast majority of the DRPs (Table 3). The most frequently referenced categorization systems were Hatoum (*n* = 5) and DOCUMENT (*n* = 3); however, 10 studies did not provide any reference to the system used (Table 3) [11,66]. A variety of assessment methods were used to categorise the clinical significance of the DRPs; most used an expert panel with a consensus approach (*n* = 8); however, some studies did not describe the assessment process (*n* = 5). The assessment of DRPs suggested that interventions in the included studies were most frequently categorised as “Moderate significant”, followed by “Minor” and “Major” (Table 3). When non-consensus methods were used for an assessment of the clinical significance, the correlation between raters was occasionally reported, and most of the studies found low correlations between the raters. Furthermore, the correlations of raters of the same profession were often low.

In addition, the number of DRPs, for which the clinical significance had been assessed, was in several studies lower than the number of DRPs identified in the study according to Table 1. Therefore, not all studies assessed all of the DRPs for clinical significance.

### 3.2. Relations between Clinical Significance and DRP Categorisation

An assessment of the relationship between the clinical significance rating and categorization of DRPs was presented for six publications at various extents [44,50,51,61,62,66].

Bondesson et al. [44] presented a cross-table of the clinical significance categories according to Hatoum [11] and DRP-categories for the 127 suggested interventions. Most of the DRPs were rated as having a “significant” clinical significance, only the ADR category had higher clinical significance ratings, and a combined category of “Wrong dosage form/wrong drug” had a lower clinical significance rating [44].

A study by Elliott & Woodward [50] also published a cross-table of the clinical significance categories according to Standards Australia and DRP-categories of 113 DRPs. A high level of clinical significance was assigned to DRPs in the categories of “Untreated indication”, “Medication management problem”, “ADR”, and “Drug interaction”, while lower levels were assigned to, e.g., “Potentially unnecessary medication” and “Inappropriate medication choice” [50].

Granaas et al. [51] used a scoring system to categorise the clinical significance by Eadon [75], and of the 388 identified DRPs, 75 DRPs were selected for a clinical significance assessment to cover all DRP categories [51]. The DRP categories with the highest clinical significance scores were “Adding a medicine”, “Drug interaction”, and “Monitoring and counselling”, while the lowest scores were assigned to “Cost-related” and “Generic substitution” [51]. ADR was not included in the DRP categorization system. Since the DRPs were non-randomly selected for the clinical significance assessment, the ratings may most likely not be generalised to the entire cohort.

In a study by Spinewine et al. [61], 700 of 1066 suggested interventions were categorised according to their clinical significance, based on van Mill and Hatoum [4,11]—366 suggested interventions were excluded, since they were assessed to have no clinical significance [61]. The categories with the highest clinical significance scores were “Change dose”, “Add a new drug”, and “Discontinue drug” [61].

Stafford et al. [62] selected the 316 DRPs with the highest clinical significance scores of 1038 DRPs using the DOCUMENT categorization system [66]. The DRP categories assigned the highest scores were “Toxicity or adverse reaction”, “Drug selection”, and “Untreated indications” [62].

Finally, Williams (2012) [66] also used the DOCUMENT categorization system to assess 5948 DRPs for clinical significance. As in the study published by Stafford et al. [62], the DRP categories assigned the highest scores were “Drug selection” and “Toxicity or adverse reaction”, but in this study, “Over or underdose” was also frequently assigned a high score [66].

## 4. Discussion

Despite the difference in the categorization systems used for DRP categorization, as well as for clinical significance categorisations, it seemed like the category “ADR” and possibly “Drug interaction” were often associated with a high clinical significance (based on six heterogeneous studies). These two categories were infrequently identified in the studies, but when identified as a DRP, they seemed to be assessed as serious for the patient. No obvious pattern between the remaining DRP categories and the level of clinical significance could be established.

### 4.1. Relations between Individual DRP Categories and Clinical Significance

An evaluation of the studies, which compared clinical significance of individual DRP categories, showed no apparent relations between the DRP categories and the level of clinical significance in general. However, “ADR”, which was one of the two categories possibly associated with a high clinical significance, has been established as a cause of hospitalization, which does support the relation with a high clinical significance [3].

Additionally, a large difference in the rating of clinical significance within individual DRP categories was observed. This may be explained by the difference in DRPs allocated to individual categories. For example, a high dose of a drug with a narrow therapeutic index may result in considerable damage to the patient compared to the prescription of a high dose of penicillin. Consequently, applying the clinical significance to individual DRP categories does not seem to provide valuable information for evaluation purposes.

### 4.2. DRP Categorization Systems

Despite a great variation in the type and number of categories of the DRP categorization systems, several papers referred to Hepler and Strand [67] regarding the choice of DRP-categories. However, the categories published by Strand et al. [68] are stated in the paper by Hepler and Strand [67], and it is likely that these categories form the basis of the majority of the DRP-categories subsequently used by other authors.

Most authors did not describe the method used to categorise DRPs. Van Mill et al. [4] reported that, in general, the validation of the categorization systems is poor. However, a practice study of a Danish DRP database showed that despite no formal training in the use of the database, the interrater reliability (Fleiss’ kappa = 0.79) and reproducibility (Fleiss’ kappa = 0.81) were high [76]. Other studies have also reported relatively high interrater reliability scores of different DRP categorization systems, with kappa values ranging from 60–75 [73,77,78,79,80]. These findings suggest that irrespective of the type of system used, clinical pharmacists seem to agree on how to categorise DRPs; however, some cases of suboptimal medication treatment may be ambiguous and hence difficult to categorise [76]. Such cases will remain difficult to categorise irrespective of the number of categories available in the DRP categorization system [76].

No single category recurred in all of the DRP categorization systems; however, some were frequently used, including the category “Other”, irrespective of the number of other categories. It is possible that this category is merely a “safe guard” to ensure that even rare and odd DRPs have a categorisation option.

The large variance in the number of categories available may be due to the type and purpose of the studies and practices locally, such as the need for the level of details, focus of medication review, patient involvement, etc. It is also possible that further categories will be added over time according to the development of medication reviews and appearance of new DRPs, e.g., related to computerised order entry systems [76,81]. Objectively, it does seems unnecessary that so many different DRP categorization systems exist with the aim of describing the findings of a clinical pharmacy service delivered internationally. International consensus on one DRP categorisation system may be impossible; however, it would improve the potential of comparing results internationally.

Finally, the clinical significance of using DRP categorization systems could be considered. For study evaluation purposes, DRP-categorisation systems are often considered as process measures to document a part of the activities delivered by the clinical pharmacist. DRP categorisation seems to be easy to apply in practice, but possibly quite time consuming. Since the ratings of clinical significance within individual DRP categories are inconsistent, the only value of applying a DRP categorization system seems to be of descriptive character. Whether the effort is worth the value should be considered for each individual study.

### 4.3. Clinical Significance Assessment

Irrespective of the number of categories available for the clinical significance categorization systems, the vast majority of the DRPs or suggested interventions were allocated into one of the following categories: “Minor”, “Moderate”, and “Major” (or similar). Whether a categorization system consisting of these three categories is optimal, will depend on the purpose of the study. Even though only a few DRPs or suggested interventions end up in the “Extremely important” and “Adverse significant” groups, these categories may serve as valuable sources for intervention purposes, i.e., to improve practice for physicians and clinical pharmacists, respectively. Some systems use a category of “No significance” [9,11], while others seem to exclude non-clinically significant DRPs. The DRPs either excluded from the evaluation or allocated to the “No significance” category may be a heterogeneous group ranging from DRPs of no clinical significance to DRPs of potential clinical significance, e.g., lack of patient understanding of medication regimen, and economical significance such as the choice of an expensive drug when an alternate inexpensive is available. The main aim of medication reviews is to optimise the medication treatment, but the cost of the drugs is often also assessed to help minimise costs for the society, as well as for the individual patient. In fact, the high cost of drugs may indirectly influence the clinical significance in the case of patients choosing to cease treatment with expensive drugs. These issues cannot be elucidated by the clinical significance categorization systems included in the current study—or, to our knowledge, any other assessment tool targeting the importance of DRPs.

The methods used to assess clinical significance included a consensus methodology. For example, expert panels may be a valid method to assess clinical significance, but using this methodology did not identify any obvious pattern between the assessments and DRP categorisation. When a consensus methodology was not used, the correlation between the raters was often low, even though only a few categories were available. This suggests that clinical significance may be related to the instruction of how to use the categorization system, but also the experience, background, and setting of the individual health professional rater. Clinical significance may even be rated differently according to the patient, who is the core person related to the medication treatment. For example, a patient might want to avoid a treatment due to side effects, irrespective of the benefits of the treatment. In addition, variation may also be a result of the variation of the type of intervention, patient population, setting, etc., of the included studies. Hence, using clinical significance categorization systems as assessment methods does not seem robust and is most likely highly dependent on the choice of individual raters. This is supported by a review by Vo et al., who found limited results for the validity and reliability of tools for assessing the potential significance of pharmacist interventions [82]. Consequently, using a clinical significance rating as a method to prioritise DRP categories does seem suboptimal. Indeed, it is possible that the low validity of DRP categorisation systems, as well as of clinical significance methods, may be a contributing factor to the fact that no apparent correlation was established.

### 4.4. Limitations

Generalisability may be questioned, since most studies were conducted at selected wards at one hospital and included less than 200 patients. However, the variation in the results of the number and types of DRPs and level of clinical significance may be explained by the heterogeneity of the studies, such as the type of intervention, training of clinical pharmacists, patient population, access to data sources, acceptance rates, etc.

Our literature search was challenged by the inconsistency of terminology used within the area of DRPs [4].” Drug related problems” (potential and actual) may be labelled differently like, e.g., “medication related problems”, and “clinical significance” may be designated, e.g., “clinical significance”. Additionally, some DRP categorization systems comprised categories of problems, while other systems used categories related to the interventions. This made a comparison of the categorization systems difficult.

As a part of presenting the data, categories were merged from the various DRP categorization systems, as well as for the clinical significance categorization systems. It is possible that the merged categories did not fully correlate with each other.

## 5. Conclusions

The current review could not establish a consistent relation between the DRP categories and the level of clinical significance. However, the categories “ADR” and possibly “Drug interaction” were often associated with a high clinical significance, albeit they were infrequently identified in the studies (based on six heterogeneous studies). Additionally, an assessment of clinical significance seemed to be a method of low validity. Hence, clinical significance ratings do not seem to be useful in prioritizing individual DRPs of the DRP categorization systems. Indeed, the value of applying DRP categorization systems to evaluate a study should be considered carefully in relation to the time spent performing the categorization. Consequently, it may be necessary to reconsider our current approach for evaluating DRPs.

## Figures and Tables

**Table 1:** Description of included studies.

Reference (Author and Year)	Country/Setting	Included Patients (pts)	Mean Age (Years)	Gender (Male)	Type of Intervention	Number of DRPs or Suggested Interventions ^1^	Acceptance Rate of DRPs ^2^	Implemen-tation Rate ^2^	Link between Clinical Significance and Type of DRPs
Alagiriswami (2009) [40]	India One hospital Medicine wards	189 pts	49.8	57.8%	Medication review	261 DRPs	87% *n* = 261	81% *n* = 261	No
Alderman (1997) [41]	Australia One hospital Acute-care, psychiatric inpatients	69 pts with DRPs	66.8	75%	Medication review	187 DRPs	92% *n* = 204	Missing	No
Blix (2006) [42]	Norway Five hospitals Six internal medicine and two rheumatology departments	672 pts with DRPs	Missing	Missing	Pharmacist contribution in therapeutic hospital team.	2128 DPRs	92% *n* = 1583	67% *n* = 1583	No
Bondesson (2013) [43]	Sweden One hospital One internal medicine ward	141 pts (70 IG, 71 CG)	81.6 years (IG: 81.9, CG: 81.3)	36% (IG: 33%, CG: 39%)	Integrated medication management	690 DRPs	93% *n* = 450	Missing	Yes
Bondesson (2012) [44]	Sweden One hospital Two internal medicine wards	132 pts	81	48%	Medication review and medication reconciliation	197 suggested interventions 127 DRPs assessed for clinical significance	90% *n* = 197	90% *n* = 197	Yes
Castelino (2011) [45]	India One hospital Renal unit	308 pts reviewed	Age groups	67.8%	Medication review	327 DRPs	97% *n* = 259	81% *n* = 259	No
Celin (2012) [46]	India One hospital Medicine and neurology wards	108 pts	Age groups	68.5%	Pharmaceutical care	80 DRPs	97% *n* = 80	88% *n* = 80	No
Chua (2012) [47]	Malaysia 44 primary care clinics	477 pts	47.9	60.2%	Pharmaceutical care	706 DRPs	Missing	87% *n* = 388	No
Elliott (2011) [48]	Australia One hospital, Aged care assessment clinic and memory disorder clinic	46 pts	82	26%	Medication history and medication review	113 DRPs	Missing	Missing	Yes
Granaas (1999) [49]	UK One general practice surgery	285 pts	65 (median)	38%	Pharmaceutical review of repeat prescriptions	IG: 90 CG: 86	IG: 86% (*n* = 90) CG: 13% (*n* = 86)	Missing	Yes
Granas (2010) [50]	Norway 23 community pharmacies	73 pts (43 pts with DRPs)	Missing	Missing	Medication review	88 DRPs	Missing	Missing	No
Kassam (2007) [51]	Canada One hospital Outpatient diabetes clinic	105 pts with DRPs	Missing	Missing	Pharmacist contribution to multidicsiplinary diabetes team	276 DRPs	Missing	Missing	No
Kumar (2013) [52]	India One hospital General medicine wards	240 pts (49 pts with DRPs)	Age groups	61.3%	Routine monitoring of patients’ medication	71 DRPs	90% *n* = 71	71% *n* = 71	No
Kumar (2012) [53]	India One hospital Medicine wards	189 pts with DRPs	49.8	57.8%	Pharmaceutical care	261 DRPs	87% *n* = 227	Missing	No
Kwint (2012) [54]	The Netherlands 10 community pharmacies	155 pts	76 (median)	46%	Home medicines review	1565 DRPs	Missing	Missing	No
Mekonnen (2013) [55]	Ethiopia One hospital Internal medicine ward	48 pts with DRPs	38	33.3%	Pharmaceutical care services including involvement in ward rounds, medication review and discharge counselling	94 DRPs	68% *n* = 149	Missing	No
Rashed (2012) [56]	UK and Saudi Arabia Two hospitals Medical ward, paediatric intensive care unit (PICU) and neonatal intensive care unit (NICU)	737 pts (333 pts with DRPs)	2.3 (median)	58.1%	Medication reivew	478 DRPs	Missing	Missing	No
Schröder (2011) [57]	Germany Community pharmacies Patients with idiopathic Parkinson’s disease	113 pts	71.50	52.2%	‘‘Drug service’’ or ‘‘pharmaceutical management’’ including medication history and medication review	331 DRPs	Missing	Missing	No
Simioni (1996) [58]	Australia One hospital Medical ward	157 pts (CG: 80, IG: 77)	Missing (CG: 68.5, IG: 69.0)	Missing (CG: 62.5%, IG: 46.8%)	Pharmaceutical care plans	IG: 154 DRPs CG: 99 DRPs	IG: 86% (*n* = 131) CG: 82% (*n* = 89)	Missing	No
Smythe (1998) [59]	USA One hospital Medical progressive care patients	287 pts (IG: 152 pts included, 131 evaluated, CG: 135)	Missing	Missing	Pharmaceutical care	818 DRPs	86% *n* = 235	Missing	No
Somers (2013) [60]	Belgium One hospital Geriatric ward	100 pts	81.4	52%	Medication review	304 DRPs	60% *n* = 304	Missing	No
Spinewine (2006) [61]	Belgium One hospital Acute geriatric unit	101 pts	82.2	27%	Pharmaceutical care from admission to discharge including participation at ward rounds	1066 DRPs	88% (+7.2% partially accepted) *n* = 1066	Missing	Yes
Stafford (2009) [62]	Australia Home-dwelling (HD) and residential care-facility (RC) patients	Missing	78.1 (HD: 73.9, RC: 83.9)	31.6 (HD: 44.2%, RC: 13.5%)	Medication reviews	1038 DRPs	Missing	Missing	Yes
Stafford (2011) [63]	Australi Community pharmacy practice	129 pts	Missing	Missing	Home medicines reviews	157 warfarin-associated DRPs	Missing	Missing	No
Stemer (2012) [64]	Austria One hospital Six different wards (1 psyciatric, 1 surgery and 4 medicine)	Missing	Missing	Missing	Clinical pharmacy service at ward rounds	478 DRPs	55% *n* = 478	Missing	No
Tejashwani Pichala (2013) [65]	India One hospital Intensive care unit	72 pts	Age groups	59.7%	Clinical pharmacy service at ward rounds	243 DRPs	47% *n* = 243	Missing	One example
Williams (2012) [66]	Australia 185 community pharmacies	Missing	Missing	Missing	Medication reviews	5948 DRPs	Missing	Missing	Some data

IG: Intervention group, CG: Control group. ^1^: When no overall number of DRPs was reported, the number of suggested interventions was included in the table instead. ^2^: Number of DRPs was used as *n*. However, if only the number of recommendations was reported, this number was used instead—or if only a limited number of DRPs were discussed with the physicians, that number was used.

**Table 2:** Categories of the DRP categorisation systems used in the included studies.

	Categorisation System Used	Untreated Indication	Improper Drug Selection	Subtherapeutic Dose	Failure to Receive Drug	Overdosage/over Dose	Adverse Drug Reaction	Drug Interaction	Drug Use without Indication	Sub-Optimal Compliance	Non-optimal Dosing	Monitoring	Medication Error	Patient Education Required	Specific Information/Therapy Discussion	Literature Search	Drug duplication/Class Duplication	Medication Management Problem	Improper Duration	Drug Use Problem/Improper Drug Use	Most Cost-Effective Agent Available	Contraindication	No specific Problem	Other	In Total
Alagiriswami (2009) *N* = 189	a	X	X	X	X	X	X	X	X															X	9
Castelino (2011) *N* = 308	a	X	X	X	X	X	X	X	X		X						XX		X					X	13
Kumar (2012) *N* = 189	a	X	X	X	X	X	X	X	X															X	9
Spinewine (2006) *N* = 101	a, h, i		X	(X) 1			X	XX	X	(X) 1	X	X	XX				X		X	XX	X		X	X	17
Alderman (1997) *N* = 69	b	X	X	X		X	X	X	X	X															8
Blix (2006) *N* = 672	b	X	X				X	X	XX	X	X	X	X	X	X	X								X	14
Elliott (2011) *N* = 46	b	X	X	X		X	X	X	X	X								X							9
Simioni (1996) *N* = 157	b	X	X	X	X	X	X	XXX	X															X	11
Bondesson (2013) *N* = 141	c	X	X	X		X	X		X	X															7
Bondesson (2012) *N* = 132	c	X	X	X		X	X		X	X															7 *
Celin (2012) *N* = 108	c	X	X	X		X	X	X	X	X														X	9
Mekonnen (2013) *N* = 48	c	X	X	X		X	X		X	X															7
Chua (2012) *N* = 477	d		X				X	X			X									X				X	6
Granas (2010) *N* = 73	d		X				X	X			X									X				X	6
Rashed (2012) *N* = 737	d		X				X	X			X									X				X	6
Kwint (2012) *N* = 155	f	(X) 1	X	(X) 1			X			X	X	X		X											7
Stafford (2009) *N* = missing	f	X	X				X			X	X	X		X										X 2	8
Stafford (2011) *N* = 129	f	X	X				X			X	X	X		X										X 2	8
Williams (2012) *N* = missing	f	X	X				X			X	X	X		X										X 2	8
Schröder (2011) *N* = 113	g		X				X	X			X									X				X	6
Stemer (2012) *N* = missing	j	X		X	X	X	X	XXXX	X			X	X		X	X				X		X		X	17
Tejashwani Pichala (2013) *N* = 72	k		XX		X		X	XX									X	X							8
Granaas (1999) *N* = 285	e	X	X	X				X			XX	(X) 3		(X) 3	X		X		X	X	X	X			13
Kassam (2007) *N* = 105	e	X	X	X		X	X	X	X	X															8
Kumar (2013) *N* = 240	e	(X) 1	X	(X) 1			X	X		X	X	X								X					8
Smythe (1998) *N* = 287	e	X					X	X	X		X	XX	X							X	X				10
Somers (2013) *N* = 100	e		X	X			X	X	X		XX									XX		X			10

The category heading covers various labels, e.g., “Untreated indication” covers, among others, “An unfulfilled indication for drug treatment”, “Need for additional drug”, “Undertreated”, “Need for additional therapy”, “Untreated condition”, “Requires drug but not receiving it”, “Lack of drug therapy”, etc. *N* refers to number of patients included. * +Categories regarding medication discrepancies, 1: Under treated/Underuse 2: Non-clinical (=e.g., alcohol, dietary or smoking problems), 3 = Monitoring or counselling. a: Modified version of Hepler and Strand [67], b: Modified version of Strand [68], c: Modified version of Cipolle, Strand and Morley [69], d: PCNE [70], e: No ref, f: DOCUMENT (based on a and d) [66], g: PIDoc [71], h: Hanlon [72], i: van Mill [4], j: Allenet [73], k: ASHP [74]. “X” indicates the presence of the category in the published categorisation system. More than one “X” indicates that more than one category of the published categorisation system falls into the category used in the current table.

**Table 3:** Clinical significance categorisations used in the included studies.

				Categories Used						
	Ref of categorization system	Assessment methods for clinical significance in current study *	No. DRP (x) or recommendations (y) where clinical significance was assessed	Extremelyimportant/Significant/life threatening/Possibly life-saving/extreme +deleterious/Type A (%)	Major/Severe/High/Very significant/possibly very important relevance/Type B (%)	Moderate/Significant/Definitely clinically significant/Medium/possibly important relevance/Type C (%)	Minor/Mild/Somewhat significant/Minimal clinical significance/Type D (%)	Low/Probably clinically insignificant/possibly low relevance (%)	Nill/No significance/Not relevant (%)	Adverse significance (%)
Alagiriswami (2009) *N* = 189	Missing	4	261	.	11	60	29	.	.	.
Alderman (1997) *N* = 69	Missing	5	187	.	20	59	21	.	.	.
Castelino (2011) *N* = 308	Alderman	5	327	.	10	16	74	.	.	.
Kassam (2007) *N* = 105	Alderman	3	276	.	31	69	0	.	.	.
Kumar (2013) *N* = 240	Alagiriswami	5	71	.	13	48	39	.	.	.
Kumar (2012) *N* = 189	Missing	1	261	.	11	60	29	.	.	.
Celin (2012) *N* = 108	Missing	5	12 ###	.	0	17	83	.	.	.
Rashed (2012) *N* = 737	Dean and Barber	3	474	.	0	27	72	.	.	.
Blix (2006) *N* = 672	Missing	2	373	6	44	40	10	.	.	.
Mekonnen (2013) *N* = 48	Missing	2	94	5	49	27	19	.	.	.
Schröder (2011) *N* = 113	van Mil	2	331	5	27	29	39	.	.	.
Bondesson (2013) *N* = 141	Hatoum	2	733 #	0.3	12	32	29	.	27	.
Bondesson (2012) *N* = 132	Hatoum	2	127y ##	0	7	51	20	.	18	3
Simioni (1996) *N* = 157	Hatoum	4	253+	0.4	14	52	16	.	18	0
Stemer (2012) ++ *N* = missing	Hatoum	4	478	0	5	38	32	.	25	<1
Chua (2012) *N* = 477	Stubbs	3	706	0.2	.	9	39	52	.	.
Elliott (2011) *N* = 46	Standards Australia	2	113	2	33	57	.	9	.	.
Granaas (1999) *N* = 285	Eadon	2	75	***	.	.	.	.	.	.
Granas (2010) *N* = 73	Missing	3	80	***	.	.	.	.	.	.
Kwint (2012) *N* = 155	Missing	4	1.565	.	42	?	.	?	.	.
Smythe (1998) *N* = 287	Missing	3	818	.	4	43	29	.	20	4
Somers (2013) ++ *N* = 100	Overhage	3	302	.	4	53	.	38	4	0.3
Spinewine (2006) *N* = 101	van Mill/Hatoum	2	334 +++	0.4 + 0.1 = 0.5	29	68	3	.	.	.
Stafford (2009) *N* = missing	Peterson	1	1.038	.	30		?	?	?	.
Stafford (2011) *N* = 129	Peterson	4	157	.	?	79	19	?	?	.
Williams (2012) *N* = missing	Peterson	1	2535	.	43		?	?	?	.
Tejashwani Pichala (2013) *N* = 72	Missing	5	192	0	17	61	22	.	.	.

#: clinical significance of the control group patients included in current table. ##: Clinical significance of the DRPs related to medication reconciliation excluded, ### Only clinical significance assessed for ADRs, * Assessment method: 1: own assessment (clinical pharmacist)—or done by more than one person, but only one assessment presented per DRP, 2: expert panel incl. consensus, 3: expert panel ÷ consensus, 4: own assessment + 1 external (or assessment solely done by 1–2 external, 5: not described, *** No sum scores were available, +: Combined intv and ctr, ++: significance levels estimated (as an average of 3 raters) presented graphically, +++: 336 DRPs of no significance excluded. *N* refers to the number of patients included.
